# Fighting Food Waste by Law: Making Sense of the Chinese Approach

**DOI:** 10.1007/s10603-022-09519-2

**Published:** 2022-06-14

**Authors:** Y. Feng, C. Marek, J. Tosun

**Affiliations:** 1 School of Government, Nanjing University, Xianlin Avenue 163, Nanjing, 210046 China; 2 Institute of Political Science, Heidelberg University, Bergheimer Straße 58, 69115 Heidelberg, Germany

**Keywords:** Food waste, Food law, Social movements, Food security, Policy development, China

## Abstract

The Standing Committee of the National People’s Congress adopted the *Anti-food Waste Law of the People’s Republic of China* in April 2021 to guarantee grain security, conserve resources, and protect the environment. We pursue three research questions: Why has China implemented a law with sanctions to reduce food waste, and why now? Why does the law target the catering industry? To answer these questions, we collected primary data through semi-structured interviews with government officials, as well as secondary data through recorded interviews available online with officials of the Legislative Affairs Commission of the Standing Committee of the National People’s Congress (NPCSC) and food waste activists, as well as NPCSC conference reports. We find a legal approach with sanctions was necessary since cultural aspects, specifically conventional Chinese dining habits and pop culture, are difficult to regulate through instruments without sanctions. In addition, we find the Chinese law focuses on the catering industry for a few reasons: (1) More waste is generated by the catering industry than households, (2) waste from the catering industry is easier to monitor than household waste, and (3) this was a response to citizen requests collected during the Anti-food Waste Law public consultation process.

## Introduction

The Sustainable Development Goals (SDGs) adopted in 2015 address food waste as one component of SDG 12 on responsible consumption and production. By 2030, Target 12.3 seeks to reduce food waste globally by 50% at consumer and retail levels, as well as reduce production and post-harvest food loss. The ethical connections between climate and food production are increasingly debated in modern society (Kortetmäki & Oksanen, 2021), with food waste being especially problematic. First of all, it contributes to environmental pollution since unconsumed food must be disposed. Second, food production generates greenhouse gas (GHG) emissions, with food surpluses creating more emissions than necessary (Liu & Nguyen, 2020). Third, food waste creates considerable economic and social costs (Morone et al., 2019). Furthermore, it is a moral issue given that millions of people worldwide lack access to adequate food and nutrition (Liu & Nguyen, 2020). With growing economic affluence and a booming catering industry, China is currently a large contributor to global food waste (Liwei et al., 2013; van den Verma et al., 2020).

In April 2021, the Standing Committee of the National People’s Congress adopted the Anti-food Waste Law, which bans excessive leftovers in restaurants. We ask why the Chinese government chose a law with sanctions as its policy response, why the law has been adopted now, and why the law targets the catering industry. To answer our questions, we collected data from semi-structured interviews conducted in June and July 2021 with Chinese government officials and a food and catering industry association, as well as recorded interviews available online and official government conference reports.

In addition, we conducted a review of China’s food waste policies since 2012 which reveals a transition from a regulatory approach without sanctions to one with sanctions following the introduction of the Anti-food Waste Law in 2021. Our interview findings reveal that international and national circumstances have influenced the initiation of China’s Anti-food Waste Law, and among these are the SDGs, COVID-19 outbreak, flooding in agricultural regions, persistent food waste, and pre-existing public policies on commodities and food. In addition, we found that unlike western countries, the Chinese law focuses on the catering industry as opposed to households. There are three reasons for this: More waste is generated by the catering industry, waste in the catering industry is easier to regulate than household food waste, and citizens requested the catering industry be targeted during public consultations on development of the Anti-food Waste Law.

## Background on the Empirical Case: the Growing Problem of Food Waste in China

Generally, discarded food can be distinguished into two categories: *food loss* and *food waste*. According to the Food and Agriculture Organization of the United Nations (FAO), the former refers to the reduction of available food during the harvesting, processing, storage, and transportation stages; the latter refers to the disposal of food, which otherwise could have been consumed, during the retail and consumption stages (FAO, 2013). In China, food loss mainly occurs due to technology and equipment limitations. In this article, we focus on food waste, which is considered a moral issue and occurs due to irrational and extravagant consumption behaviour (Blair & Sobal, 2006). There are three dining settings where food is wasted in the consumption stage: households, restaurants, and canteens (including those of schools, hospitals, government departments, etc.).

The catering industry in urban China has been critiqued for generating exorbitant food waste (Li et al., 2021; Liwei et al., 2013; Wang et al., 2017), with discussion of a food waste crisis in China during recent years (Li et al., 2019, 2021; Liu et al., 2016; Liwei et al., 2013; Min et al., 2021; Song et al., 2018; Xu et al., 2020). There is even a collection of literature on food recycling and the extraction of energy from restaurant food waste in China (Clercq et al., 2016, 2017; Lang et al., 2020; Yang et al., 2019; Zhang et al. 2019). Food waste appears to increase when consumers dine more outside of the household (Pinpart et al., 2019), as well as with increasing consumer income, a phenomenon termed “affluence elasticity of waste” (van den Verma et al., 2020). In China, both trends are present. Consumers are dining out more often (Liwei et al., 2013, p. 338; Xu et al., 2020), with an estimated 3.5 million catering companies in China including large, medium and small restaurants, snack and fast-food outlets, and cafeterias (Feng, 2019), and rapid economic growth during the past three decades (World Bank, 2020).

Many studies have quantified food waste in various Chinese dining settings (Cao & Li, 2009; Liu et al., 2013; WWF & IGSNRR, 2018; Zhang & Fu, 2010). A survey from WWF found the per capita food waste rate in China’s catering industry to be as high as 12% of all food served (WWF & IGSNRR, 2018). Further studies have estimated China’s share in global food waste to have increased from just 5% in 1992 to 27% in 2013 (Lopez Barrera & Hertel 2021, p. 101,879). It has also been estimated that the food waste in four major Chinese cities during 2015 alone could have fed 30–50 million people in one year, roughly the population of South Korea at that time (Liu, 2018). The United Nations Environment Program’s Food Waste Index Report, 2021 shows that Chinese households produce 64 kg/capita food waste per year as compared to 59 kg/capita food waste per year in the USA (United Nations Environment Programme, 2021). Regarding wasted resources, researchers estimate 135 billion m^3^ water and 26 million ha cropland were used to produce China’s wasted food in 2010 alone (Liu et al., 2013).

Table 1 provides an overview of food waste estimates for China during the consumption stage. Although food waste is quantified differently between studies, it is apparent that restaurants and school canteens account for a large part of food waste in China, which is different from other developed countries where the majority of food waste is generated at home (Aschemann-Witzel et al., 2019; Gooch et al., 2010; Herzberg et al., 2020; Kasza et al., 2020). The United Nations Environment Programme (UNEP) has found in a worldwide survey that 61% of food waste comes from households (United Nations Environment Programme, 2021). While UNEP estimates 16 kg/capita food waste per year in the retail sector in China, they have admitted very low confidence in their estimate, which could mean that actual capita food waste/year in the retail sector is higher than current best estimates (United Nations Environment Programme 2021; Database).

**Table 1:** Estimated food waste in China during recent decades

Reference	Region	Year	Food waste		
			Restaurant	Canteen	Household
Cao & Li (2009)	Henan	2008	18.63% of food served	5.42% of food served	7.34% of food served
Zhang & Fu (2010)	Beijing	–	290 g/cap/meal	Colleges: 360 g/cap/day	70 g/cap/day
Liu. et al. (2013)	Nationwide	2010	Grain: 19% of food served	Grain: 5% of food served	Grain: 7% of food served
WWF & IGSNRR (2018)	Beijing, Shanghai, Chengdu, Lhasa, etc.	2015	12% of food served, 93 g/cap/meal	Schools: 21% of food served, 130 g/cap/meal	–
China Agricultural University (2011)	Nationwide	2007–2008	Protein: 8 billion kg Fat: 3 billion kg	–	–
Song et al. (2015)	Nationwide	2011	–	–	44 g/cap/day
Li et al. (2021)	Shandong	2017	–	–	Rural: 8.74 g/cap/meal
Wang et al. (2017)	Beijing, Shanghai, Chengdu, Lhasa	2015	93 g/cap/meal	–	–
Zhang et al. (2020)	Henan	2018	–	–	Urban: 5.54 g/cap/meal

## Theoretical Considerations on the Emergence of Food Waste Policy in China

To provide a more general theoretical argument, we draw from the notion of policy styles, which was first put forth by Richardson et al. (1982). This concept contends there are entrenched and, therefore, stable ways of policymaking and implementation in countries. To characterize national policy styles, Richardson et al. (1982) differentiate between anticipatory and reactive styles. A reactive style implies governments respond to issues as they emerge, and an anticipatory style is attributed to governments that collect information and knowledge in an attempt to address issues before they become virulent. A second dimension of policy styles is whether governments impose public policies on societal actors or whether they negotiate and bargain with societal actors. Considering the concentration of power in China’s political leadership, and the significant leeway bureaucrats have in public policy implementation, it is reasonable to expect the Chinese policy style is reactive (as local information is in short supply given very limited political participation) and imposes policies on societal actors. However, as Qian (2018, 2021) compellingly demonstrates, Chinese policymakers do respond to bottom-up inputs and can be anticipatory in their policy style.

Chinese social media platforms provide policymakers with citizen opinions and allow environmental issues to be relatively openly debated (Rauchfleisch & Schäfer, 2015). While the government remains selective in its responses and focuses on short-term gains (Tang et al., 2018), citizen interest groups are able to influence environmental policymaking in China through public campaigning which places pressure on policymakers to act (Popović, 2020). In addition, bottom-up societal pressure through government channels, such as online public consultation processes, can motivate government responsiveness in China (Chen et al., 2016). Public consultations have become common in policymaking during recent years, where the Chinese central government has indeed been responsive to citizen requests (Kornreich, 2019). However, responsiveness to Chinese public opinion remains a strategic move of the central government to reinforce its legitimacy and maintain hegemony in governing national matters (Li et al., 2016b; Noesselt, 2014). By remaining responsive, the central government is able to maintain control of both policy development and public approval of the Central Party Committee (CPC).

Following research on the motives of China’s responsive governance strategy (Li et al., 2016b; Noesselt, 2014), we expect to see a causal connection between the journalist Zhijun Xu’s initiation of the “Clean Your Plate” campaign on Weibo and the Chinese civil society group IN_33’s continuation of this online campaign, with the release of the Anti-food Waste Law in China in April 2021. We also expect to observe a causal connection between citizen requests on the defining characteristics of policy for addressing food waste and the Chinese government’s final Anti-food Waste Law. Given China’s openness to the debate of environmental issues (Rauchfleisch & Schäfer, 2015) and its responsiveness to citizen interests via social media and public consultation processes (Kornreich, 2019; Li et al., 2016b; Noesselt, 2014), it is reasonable to anticipate that the Chinese government will justify its Anti-food Waste Law based on citizen requests and bottom-up societal pressure (Chen et al., 2016).

## Methodology

This paper first presents policies relevant for controlling food waste implemented during the last decade to explore the emergence of food waste policy in China. The main sources for identifying relevant policies were the Chinese central government’s official website, and the PKULaw website, which offers legal materials from the People’s Republic of China (www.pkulaw.com). We used the terms “food waste” and “food saving” to search this comprehensive database. To ensure the relevance of the collected policy data, we applied three further criteria. First, the main content or body of the policy had to be directly related to food saving or food waste; second, the issuing departments had to be limited to the National People’s Congress and its Standing Committee, the State Council, and the Central Committee of the Communist Party of China; and third, the level of authority had to be limited to laws, regulations, and normative documents. Following these criteria, 10 relevant policy documents issued from 2012 to 2021 were collected and assessed.

To explain how the Anti-food Waste Law came about, we collected primary data through interviews with government officials from provincial and county levels over WeChat and telephone. We posed our three research questions during the expert interviews, which lasted from 20 min to over an hour. We cross-validated answers through multiple interviews and asked interviewees to provide examples based on their work experience. Secondary data were collected through archived recorded interviews with officials of the Legislative Affairs Commission of the Standing Committee of the National People’s Congress (NPCSC) and food waste activists, as well as reports from NPCSC conferences available online. We conducted semi-structured interviews in June–August 2021 with nine individuals: eight government officials and a representative of a food and catering industry association. We focused on three key government departments when selecting our interview partners: first, the Administration for Market Regulation, which is responsible for food safety regulation (production, sales, and catering) within respective administrative regions; second, the Bureau of Commerce, which regulates the Chinese catering industry; and third, the Food and Strategic Reserves Administration, which is responsible for monitoring grain and material reserves to ensure national food security. The final organization we interviewed was the Food and Catering Industry Association, which is responsible for implementing national laws and representing the common interests of the catering industry. The list of anonymized interviewees is provided in Table 2.

**Table 2:** List of interviewees

Interviewee	Affiliation	Date	Tools
A	Administration for Market Regulation	June 21, 2021	WeChat
B	Administration for Market Regulation	June 21, 2021	WeChat
C	Administration for Market Regulation	June 22, 2021	WeChat
D	Administration for Market Regulation	July 12, 2021	WeChat
E	Administration for Market Regulation	July 26, 2021	WeChat
F	Administration for Market Regulation	August 1, 2021	WeChat
G	Bureau of Commerce	June 26, 2021	Telephone
H	Food and Strategic Reserves Administration	June 22, 2021	WeChat
I	Food and Catering Industry Association	June 23, 2021	WeChat

In addition, we collected and analysed online interview coverage with Zhijun Xu (Song, 2021), one of the initiators of the Clean Your Plate campaign, and members of the Legislative Affairs Commission (Xu, 2020b). Lastly, we analysed official conference reports which document the drafting process of the Anti-food Waste Law and its open public consultation process. Following legislative records (NPC Observer, 2021), we identified and analysed four conference reports on the Anti-food Waste Law: (1) *Investigation Report on Cherishing Food and Anti Waste* from the special research group of NPCSC (Wu, 2020), (2) *Explanation on the “Anti-food Waste Law (draft)”* from the Legislative Affairs Commission of NPCSC (Xu, 2020a), (3) *Report on Results of Deliberation*, and (4) *Report on Suggestions for Revision* from the Constitution and Law Committee of the NPC.

## Development of Chinese Public Policy on Food Waste

Although the interest in food waste reduction in China during the past decade appears to be sudden, the Chinese central government introduced many anti-food waste policies during the past century. After the founding of New China in 1949, Mao launched a movement against the “three evils” (corruption, waste, and bureaucracy) in 1951. Given two distinct approaches in China’s anti-food waste strategies, anti-food waste policies in China can be grouped into two phases: (1) 1949 to 2011 and (2) 2012 to present.

### First Phase: Government-Led Conservation of Food (1949–2011)

Chinese food policy in the early twentieth century was focused on food security, rather than food safety or food quality. From 1949 to 1978, the Chinese food supply was regulated based on a “planned quantity and even distribution” model with food stamps to regulate supply shortages. From 1979 to 2011, national food production increased gradually, and the food supply began to stabilize (National Bureau of Statistics, 2020a). The planned control of food consumption was abolished in 1993. Studies on food loss and waste emerged during the following few years, with one study calculating overall post-harvest waste at 18% due to faulty technology (Guo, 2015). However, food waste began to emerge as a policy issue in 2002 when the *State Council Circular to Further Enhance Grain Saving and Food Waste Reduction* developed detailed measures on assessing food loss and waste reduction in China. While grain production in China stabilized to above 400 kg per capita in 2008, a global food crisis arose (Zhao, 2020), leading to the release of China’s *Outline of the Programme for Mid-and Long-term National Grain Security (2008–2020)*, which calls for food waste reduction.

### Second Phase: Exchange Between Government and Civil Society to Address Food Waste (2012–Present)

While food waste emerged in Chinese public policy during the early 2000s, the issue has only received large-scale attention during the past decade, with China’s governing approach shifting from moral restraint to sanctioning food waste. In 2012, China’s focus on domestic political corruption and the Clean Your Plate campaign created a policy window for addressing food waste (Kingdon, 2014). In early 2012 before the campaign, the Agricultural Law (2012 revision) simply promoted that food be cherished and saved without repercussions for violators (Table 3). The new Chinese government formed in 2012 issued the *Eight-point Regulation* and *Six-point Ban*, which aimed to curb lavish government banquets generating excessive food waste. Here, requests from President Xi were set forth, but again without negative consequences for violations. In early 2013, Chinese civil society launched a bottom-up campaign that quickly spread over Weibo. Meanwhile, food waste continued to gain prominence in Chinese public policy, with the *Regulations of Party and Government on Practicing Thrift and Opposing Waste* in 2013 and the *Opinions on Practicing Thrift and Opposing Food Waste* released in 2014. Here again, goals are set without consequences for violation. In all further relevant policies leading up to the Anti-food Waste Law in 2021, a similar pattern is present with policy instruments promoting awareness of food waste reduction and “greener” behaviour among citizens after the SDGs were introduced in 2015. Notably in 2016, China vowed to reduce its food loss and food waste rate by over 40% in 2020 (National Development and Reform Commission, 2016) and mentioned the promotion of the Clean Your Plate campaign in its *Work Plan for Greenhouse Gas Emission Control During the 13*^*th*^* Five-Year Plan Period*. Table 3 provides an overview of all relevant policies in China addressing food waste from 2012 leading up to the present with the introduction of the Anti-food Waste Law. In general, these policies gradually evolved from regulatory policies employing moral restraint to sanctioning behaviour resulting in food waste (for a general overview on legal approaches, see Snyder (1993).

**Table 3:** Laws, regulations, and policies about food waste in China (2012–2021)

No	Laws, regulations, and policies	Relevant content	Issuing date	Issuing division	Policy instruments and sanctions	Targeted actors
1	Agricultural Law (2012 Revision)	“The State promotes the cherishing and saving of food and takes measures to improve the nutritional structure of people’s food”	2012/02/28	NPC	Nodality: no sanctions	General public
2	Eight-point Regulation and Six-point Ban (a series of documents)	A campaign against official extravagance and governmental reception meals at public expenses Follow-up documents stated: violation of these regulations/bans will be held accountable in accordance with the Regulation of the Communist Party of China on Disciplinary Actions	2012/12/04	Politburo	Authority: Party discipline penalty	Officials
3	Regulations of Party and Government on Practicing Thrift and Opposing Waste	“No banquets during the meetings of party committees and governments” “All propaganda departments should take ‘practicing thrift and opposing waste’ as an important content” “Party committees and governments at all levels should establish a supervision and accountability system for ‘practicing thrift and opposing waste’.” “Those officials who violation of the rules should be dealt with severely, and those should be publicly exposed in the event of aggravating circumstances”	2013/11/18	CPC and SC	Nodality & authority: Party discipline penalty & administrative penalty	Officials
4	Opinions on Practicing Thrift and Opposing Food Waste	Detailed countermeasures on eliminating official meals waste, promoting scientific and civilized food consumption patterns, reducing food loss and waste in all sectors, and promoting the resource utilization of food waste “For violations of official reception regulations and banquets with public funds, relevant personnel shall be investigated for responsibility according to discipline and law, and the main officials in charge or relevant leaders with leadership responsibility shall be held accountable”	2014/03/11	CPC and SC	Nodality & authority: Party discipline penalty & administrative penalty	Officials Catering industry Canteens
5	Opinions on Accelerating the Advancement of Ecological Civilization Construction	“Carry out green life campaign extensively…… firmly resist and oppose all forms of extravagance, waste and unreasonable consumption.” “Launch an all-round anti-food waste action in catering enterprises, canteens, and families”	2015/04/25	CPC and SC	Nodality: no sanctions	General public Catering industry Canteens
6	Outline of the 13th Five-Year Plan for the National Economic and Social Development	“Advocate reasonable consumption while opposing waste and extravagance. Work to see that economy is practiced throughout all stages—from production to distribution, storage, and consumption. Exercise effective control over the abuse of public funds, take action against over-packaging, food waste, and overconsumption, and work to see that frugality becomes a social norm”	2016/03/16	NPC	Nodality: no sanctions	General public
7	Work Plan for Greenhouse Gas Emission Control during the 13th Five-Year Plan Period	“Promote low-carbon dining, promote ‘clean your plate’ campaign and curb food waste”	2016/10/27	SC	Nodality: no sanctions	General public
8	Opinions on Comprehensively Tightening Ecological and Environmental Protection and Resolutely Fight for the Uphill Battle for Prevention and Control of Pollution	“Strengthen the publicity and education of ecological civilization, encourage simple, moderate, green, and low-carbon ways of life, and oppose extravagance and excessive consumption”	2018/06/16	CPC and SC	Nodality: no sanctions	General public
9	Several Opinions on Improving and Promoting the Consumption System and Mechanism and further stimulating consumption	“Raise awareness of green consumption in the whole society, encourage simple, moderate, green, and low-carbon ways of life, abstain from extravagant consumption and unreasonable consumption, and promote sustainable consumption”	2018/09/20	CPC and SC	Nodality: no sanctions	General public
10	Anti-food Waste Law	Detailed countermeasures on practicing thrift and opposing waste, including posting or placing anti-food waste signs at conspicuous places and direct diners to ordering and taking an appropriate amount of food, organizing anti-food waste publicity and education, as well imposing penalties on officials, catering industry, canteens, and video service providers	2021/04/29	NPC	Nodality & authority: administrative penalty	Officials General public Catering industry Canteens Video service providers

*SC* State Council, *NPC* National People’s Congress, *Politburo* political bureau, *CPC* Central Party Committee

### Emergence of the Anti-food Waste Law

Perhaps surprisingly, the direct events leading to China’s Anti-food Waste Law were partly initiated by a social movement (Song, 2021). On April 22, 2012 (Earth Day), journalist Zhijun Xu posted a photo of an empty plate on Weibo, with the caption “Operation Empty Plate” (Magistad, 2013). This did not arouse public attention at the time, but the campaign gained wide recognition in January 2013 from the group IN_33, which advocated to “start with me, no leftovers on the plate today” (Pokojski, 2015). The group was motivated to start the Clean Your Plate campaign after observing student food waste on college campuses (Magistad, 2013). To raise awareness, IN_33 campaigned on Weibo against food waste and distributed leaflets and posters in Beijing restaurants and gas stations (Dong, 2013b; Mirosa et al., 2018).

Importantly, IN_33’s call to action came just after President Xi came to power. At the start of his presidency, Xi demanded an end to extravagant banquets and other self-indulgences by all government officials, issuing the *Eight-point Regulation* on December 4, 2012. In January 24, 2013, followers on Weibo had posted 13,795,497 microblogs contributing to China’s anti-food waste discourse (Dong, 2013a). On January 17, 2013, President Xi acknowledged the Clean Your Plate campaign, remarking: “These wasteful habits must stop immediately!” (Sui et al., 2013). A Xinhua News Agency report released a written comment by President Xi that food waste in official receptions was strongly criticized by the public (Sui et al., 2013). However, the Chinese public was already aware of food waste among public officials in 2011, which was shown through a public poll titled “Which aspects of society are most wasteful?,” with 93% of the 2707 respondents choosing “banquets at public expenses” (Xiao, 2011). With official media such as *The People’s Daily* and public figures paying close attention to the campaign, the anti-food waste movement gained legitimacy and traction in Chinese public policy, enabling the growing social movement and the Chinese government to work together on food waste reduction.

Following the Clean Your Plate campaign focused on government officials, the catering industry in Shanghai and Beijing saw their business drop by as much as 35% (Keating, 2014; Magistad, 2013). The campaign clearly had impact, but a survey revealed Chinese consumers still wasted roughly 17–18 million tons of food in big cities in 2015 (WWF & IGSNRR, 2018). In August 2020, central government departments, local governments, and catering associations launched a second Clean Your Plate campaign for the general public to address food waste in direct response President Xi’s speech on August 11, 2020, emphasizing that COVID-19 had “sounded the alarm” on food waste, and the country must remain vigilant about its food security (Xinhuanet, 2020a). This concern is very legitimate, seeing as COVID-19 had significant impacts on food access even in the most developed nations (Oncini, 2021). Within 3 days, the Clean Your Plate campaign (#GuangPanXingDong) generated around 870,000 posts and 550 million views on Weibo (Xinhuanet, 2020b). The anti-food waste legislation process began directly in August 2020 and was officially concluded with the final Anti-food Waste Law in April 2021.

### Summarizing Trends in Food Waste Policy Development in China

According to Hood and Margetts (2007), policy instruments can be categorized into four groups based on the government sources used to implement them: nodality, authority, treasure, and organization (Hood & Margetts, 2007). This is known collectively as the *NATO* policy instrument typology as has recently been applied to the analysis of agri-food policy (e.g., Bazzan et al. (2022). Nodality and treasure-based instruments are indirect and voluntary measures, which are not usually backed by sanctions in the case of food waste policy in China, unless they are combined with authority instruments (discussed more below). Examples of nodality instruments include information campaigns such as promotion of the Clean Your Plate campaign, persuasion (nudging), and advocacy for reducing food waste (for example, to meet sustainability goals). Treasure instruments include monetary rewards for reducing food waste, but the target group is not obligated to follow the rules.

By contrast, authority instruments are non-voluntary and directly affect the policy’s target group. These instruments correspond to regulatory approaches with sanctions, which, in the case of food waste policy in China, tend to be either Party or administrative sanctions. Party sanctions are targeted at members of the CPC who violate Party disciplines and, according to the *Regulation of the Communist Party of China on Disciplinary Actions (2018 Revision)*, include warning, serious warning, removal from Party position, be placed on probation within the Party, and expulsion from the Party. Administrative sanctions are penalties against illegal misconduct of state civil servants or affiliated personnel according to the *Civil Servant Law of the People’s Republic of China (2018 Revision)* and include warning, demerit, gross demerit, demotion, removal from office, and dismissal. Administrative penalties against a citizen, legal person, or another organization for violation of the administrative order according to the *Law of the People’s Republic of China on Administrative Penalty (2021 Revision)* include (1) warnings and circulation of criticism; (2) fines, confiscation of illegal gains, and confiscation of illegal property; (3) temporarily detaining the license, lowering the qualification level, and revoking the license; (4) restricting the development of production and business operation activities, ordering suspension of production and business, ordering closure, and restricting employment; (5) administrative detention; and (6) other administrative penalties as prescribed by laws and administrative regulations.

The Anti-food Waste Law is an example of an authority instrument with sanctions, since non-compliance leads to negative consequences, and all members of the policy target group are required to comply. Furthermore, the *Eight-point Regulation*, the *Six-point Ban*, *Regulations of Party and Government on Practicing Thrift and Opposing Waste*, and *Opinions on Practicing Thrift and Opposing Waste* are also authority instruments (some are mixed nodality/authority instruments, see Table 3), which all put forth negative repercussions through sanctions for officials who generate food waste. Organization tools take neither direct nor indirect action on a target group; rather the government itself assumes responsibility for providing a public service. For our case, we did not find any examples of these tools. Before the release of the *Anti-Food Waste Law*, nodality instruments (without sanctions) were implemented to encourage the general public to save food and prevent food waste, while authority instruments (with sanctions) were implemented to control government officials’ food waste and address online food sales (Snyder & Kim, 2018).

We see the emergence of authority-based instruments with sanctions in countering food waste among the general public in China with the introduction of the Anti-food Waste Law in April 2021. Unlike previous policies, this law imposes clear administrative penalties (warnings and fines) on those wasting food (Table 4), such as food service providers if they mislead consumers to order excessive meals. Interestingly, the penalties are directly targeted at commercial entities, such as the catering industry and live-streaming entertainment companies, while the general public is indirectly targeted, and fines for food waste can be passed to customers by restaurants.

**Table 4:** Overview of the penalty conditions and measures of the Anti-food Waste Law

Targeted actors	Examples	Penalty conditions	Penalty measures
Catering service providers	Restaurants, snack bars, beverage shops, etc	Fail to actively remind consumers to prevent food waste	**Warning:** take corrective action and be warned
		Mislead consumers to order excessive food and cause evident waste	**Warning:** take corrective action and be warned **Fine:** if the violator refuses to take corrective action, they shall be fined between 1,000 and 10,000 Yuan
Food producers or dealers	Food production and processing enterprises, food retailers, catering industry, canteens, etc	Cause serious food waste in food production and trade	**Fine:** take corrective action, and if the violator refuses to take corrective action, they shall be fined between 5,000 and 50,000 Yuan
Entities with canteens	Schools, hospitals, enterprises, etc	Fail to formulate or implement measures to prevent food waste	**Warning:** take corrective action and be warned
Radio stations, TV stations or network audios, and video service providers	Radio stations, TV stations or network audios, and video service providers	Produce, release, or disseminate programmes or audio and video information advocating excessive food consumption and gluttony	**Warning:** take corrective action and be warned **Fine:** if the violator refuses to take corrective action or the circumstances are serious, they shall be fined between 10,000 and 100,000 Yuan **Business suspension:** and may be ordered to suspend relevant business or cease business operation for an overhaul **Other sanctions:** and the directly responsible executive in charge and other directly liable persons shall be subject to legal liability in accordance with the law

## Analysis

### Explaining the Transition Towards Law with Sanctioning Power

From our analysis of food waste policies in China, we see that the Anti-food Waste Law was, in fact, not a sudden development. There were many prior policy instruments directed at the general public to regulate food waste. However, in 2013, we see the first sanctions introduced for countering food waste by regulating overconsumption at official government banquets. These policies paved the road for implementing the new Anti-food Waste Law for targeting consumers, and the catering and media industries. Given the observed evolution of policy tools, and the inability of prior policy instruments to significantly reduce food waste, we interpret that sanctions were necessary for controlling consumer food waste in China.

The policy approach adopted initially by the Chinese government was unable to effectively control food waste and consumer behaviour/culture aspects (Qiang, 2021). Without interventions with sanctioning power, there was not motivation to change embedded cultural practices. Cultural aspects, such as “face saving,” have been found to significantly influence diners’ habits, reducing the likeliness that individuals will pack their leftovers and thereby reduce food waste in restaurant settings (Liao et al., 2018). Saving face and appearances have also been confirmed in our interviews as a key challenge in addressing food waste at large events (Feng, 2021). On the other hand, Liao et al. (2018) find that the cultural aspect of “group conformity” can positively impact behaviour when ordering smaller portions is accepted and practiced among an individual’s dining company.

**Fig. 1:**
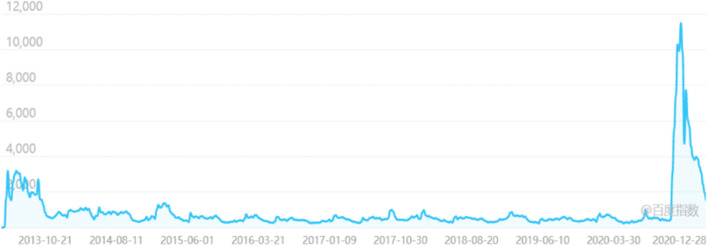
Baidu search index “Clean Your Plate” campaign (2013–2020)

As the two Clean Your Plate campaigns in China occurred following civil society initiative, we find that civil society has been a driving factor in shifting the focus of authority instruments with sanctioning power for food waste reduction from targeting government officials to targeting consumers. Although the food waste movement advocated waste reduction and cultural change through social media campaigning, and not regulatory measures, we find that without this awareness-raising, individuals surveyed in the public consultation process likely would not have pushed for a law with sanctions targeting the catering industry. We can see a strong increase in the awareness of food waste reduction based on increased searches for the Clean Your Plate campaign in 2013 at its initial start, as well as in 2020 after the government launched the second campaign during the COVID-19 pandemic (Fig. 1). According to one of the Clean Your Plate campaigners, social concern for the campaign increased after the introduction of the *Eight-point Regulation*, with many influential people following the movement too (Song, 2021). Measures first promoted by the Clean Your Plate campaign, such as restaurants actively reminding consumers to prevent food waste and serving smaller portions, are now becoming articles of the Anti-food Waste Law (NPCSC, 2021).

### Explaining the Timing of the Chinese Anti-food Waste Law

**Fig. 2:**
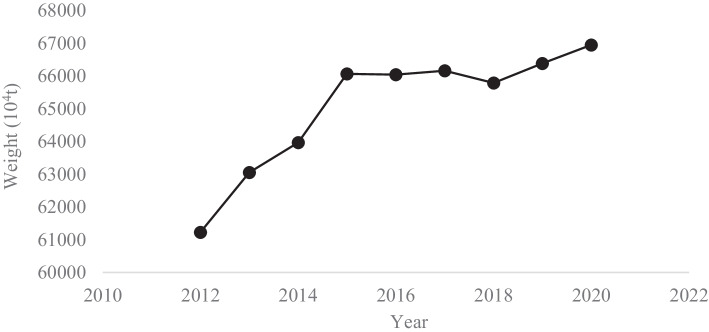
Output of farm productions in China (2012–2020). Data source: China Statistical Yearbook (2018–2020)

The timing of the Anti-food Waste Law is especially interesting, as China is mostly food secure, and its agricultural production has increased during the past decade (National Bureau of Statistics, 2018, 2019, 2020) (Fig. 2). In 2020, China’s total food output was 669.49 million tons, and per capita food production in 2019 reached 474.95 kg (National Bureau of Statistics, 2020b). The white paper “China’s Food Security” released in 2019 shows that the self-sufficiency rate of cereals is over 95%, with rice and wheat at over 100% self-sufficiency (The State Council Information Office of the People’s Republic of China, 2019).

**Fig. 3:**
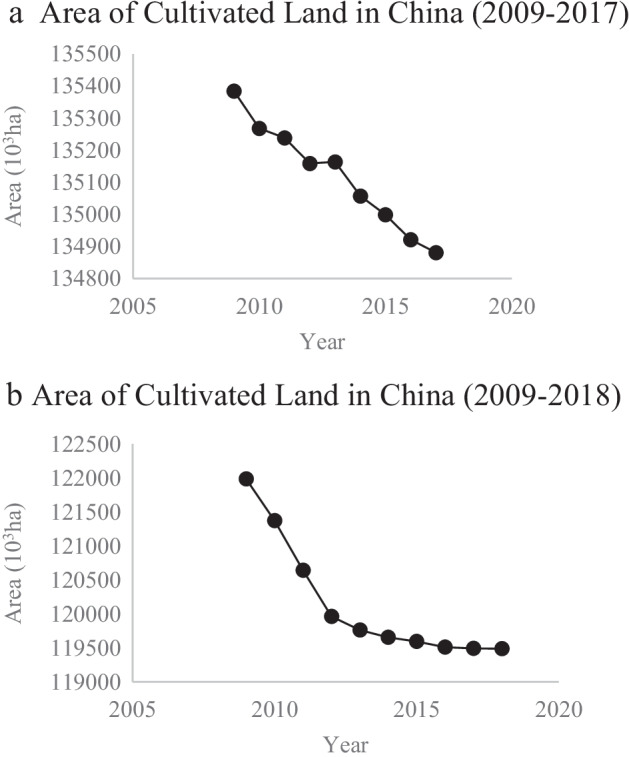
**a** Area of the cultivated land in China (2009–2017). Data source: China Statistical Yearbook (2015–2019). **b** Area of the cultivated land in China (2009–2018). Data source: The Word Bank (https://databank.worldbank.org/reports.aspx?source=world-development-indicators)

However, despite increasing agricultural production, the area of cultivated land in China declined from 2012 to 2017 and in 2019 (Fig. 3a, b). According to the Bureau of Commerce (RZH, county level), there are many challenges in securing domestic food supply, including low agricultural subsidies, which do not encourage farmers to produce food, as well as China’s preference to import food, land degradation, increasing urbanization, land abandonment in the countryside, and China’s large population (Interviewee G, 6/26/2021). These challenges have now reached a “boiling point” leading to the introduction of the Anti-food Waste Law in 2021 (Interviewee G, 6/26/2021). In addition, addressing food waste enables China to contribute to the SDGs and achieve carbon neutrality by 2060 (Interviewee E, 7/26/2021).

We found through our research that the COVID-19 pandemic, and the worst floods since 1998 in major rice production areas of China, triggered a sense of urgency for addressing food security and food waste reduction. Twenty-two countries responded to COVID-19 with food export restrictions (Hepburn et al., 2020), while China had record numbers of agricultural imports in 2020 (General Administration of Customs of the People’s Republic of China, 2020). According to official statistics, food prices rose about 13% in July 2020 compared to the prior year, and the price of pork, a staple food for many Chinese families, increased by about 85% during the same period (National Bureau of Statistics, 2020c). Within two days after the release of these statistics, the second Clean Your Plate campaign was launched (Fu & Ren, 2020). Thus, the Chinese government’s concern for national food security during the COVID-19 pandemic led to President Xi’s concern for food waste.

### Explaining the Anti-food Waste Law’s Focus on the Catering Industry

The Anti-food Waste Law targets dining behaviour outside of the household, because most waste occurs in public settings. This has been shown through our literature review as well as our interviews (Interviewee D, 7/12/2021). The catering industry provides an optimal environment for regulating food waste, as personal food waste at home is difficult to monitor and discourage. As part of the policy development process, two public surveys were conducted. According to the survey report of the Standing Committee of the National People’s Congress (1^st^ public survey), 75% of the respondents believe that waste in the consumption stage is most serious. Business banquets (42%), public funds consumption (27%), school canteens (14%), and family and friends’ gatherings (12%) were considered the main food waste settings (Wu, 2020). Results from the public consultation process on the draft law showed the general public wanted the catering industry to be held responsible for its contribution to food waste (National People’s Congress, 2021).

## Conclusion

This study contributes an interesting perspective to the literature by documenting the initiation of food waste legislation in China. Our study revealed that the NPCSC did not opt for a law with sanctions on food waste as its first action, but rather that this approach was chosen after prior policies without sanctions were unsuccessful. It is particularly interesting that civil society demands resulted in the adoption of the law. However, in accordance with the existing literature (e.g., Li et al., 2016a), our study shows that the NPCSC responds to policy demands from subnational units or the public if these concur with its strategic goals. This has the advantage for the political regime that it can legitimize its policy actions (Chen & Xu, 2017), which is particularly important when adopting laws with sanctions. Concerning the timing of the ban, we could identify several factors that mattered such as the COVID-19 pandemic, and its implications for food production in China and the countries from which China imports food. It is interesting to note that sanction instruments, like warnings and fines, in the Anti-food Waste Law mainly focus on the catering industry. We found this to be due to more waste being generated by the catering industry than households, and because food waste is easier to monitor in public settings. In addition, a focus on the catering industry in the Anti-food Waste Law was in response to requests from the general public. We would argue that the perspective of civil society was considered, because it represents a functionally meaningful approach since it is easier to regulate and monitor the behaviour of the catering industry than personal food waste.

The objective of this study was to explain how the Anti-food Waste Law came about and to demonstrate that policymaking in China, an authoritarian state, is characterized by an incremental approach as in democracies (Walder, 2015). This insight contributes to the small but growing literature on policy design (Capano & Howlett, 2021; Chindarkar et al., 2017; Howlett & Mukherjee, 2014). In a complementary fashion, this study provides insight into policy-oriented learning in authoritarian regimes, which has tended to focus on how China learns from abroad (Ortmann & Thompson, 2014; Snyder, 2016). However, less attention has been paid to how Chinese policymakers draw lessons from their previous attempts to address an issue. Our study provides valuable insights for learning more about inter-temporal policy learning, as well as political learning in the strategies adopted by policymakers (Bennett & Howlett, 1992; Dunlop & Radaelli, 2020).

Further research could track the implementation of the Anti-food Waste Law in China and explore the effectiveness of sanctions in reducing food waste caused by cultural practices. Concerning implementation, in other policy areas, China has suffered from deficits due to the relationship between the central and local administrative levels (Qi & Zhang, 2014; Yasuda, 2018). Further research could also investigate to what extent the Chinese policy approach to food waste will be followed by other countries: authoritarian, democratic, or both. Such a perspective is typically adopted by research on transnational policy diffusion processes. If applying this theoretical lens, the policy approach adopted in China would be considered a policy innovation, and it could be asked whether this policy innovation diffuses due to its existence or due to its demonstrated effectiveness.
